# Identifying HIV-induced subgraph patterns in brain networks with side information

**DOI:** 10.1007/s40708-015-0023-1

**Published:** 2015-11-16

**Authors:** Bokai Cao, Xiangnan Kong, Jingyuan Zhang, Philip S. Yu, Ann B. Ragin

**Affiliations:** 1 Department of Computer Science, University of Illinois at Chicago, Chicago, IL USA; 2 Department of Computer Science, Worcester Polytechnic Institute, Worcester, MA USA; 3 Institute for Data Science, Tsinghua University, Beijing, China; 4 Department of Radiology, Northwestern University, Chicago, IL USA

**Keywords:** Subgraph pattern, Graph mining, Side information, Brain network

## Abstract

Investigating brain connectivity networks for neurological disorder identification has attracted great interest in recent years, most of which focus on the graph representation alone. However, in addition to brain networks derived from the neuroimaging data, hundreds of clinical, immunologic, serologic, and cognitive measures may also be documented for each subject. These measures compose multiple side views encoding a tremendous amount of supplemental information for diagnostic purposes, yet are often ignored. In this paper, we study the problem of subgraph selection from brain networks with side information guidance and propose a novel solution to find an optimal set of subgraph patterns for graph classification by exploring a plurality of side views. We derive a feature evaluation criterion, named gSide, to estimate the usefulness of subgraph patterns based upon side views. Then we develop a branch-and-bound algorithm, called gMSV, to efficiently search for optimal subgraph patterns by integrating the subgraph mining process and the procedure of discriminative feature selection. Empirical studies on graph classification tasks for neurological disorders using brain networks demonstrate that subgraph patterns selected by the multi-side-view-guided subgraph selection approach can effectively boost graph classification performances and are relevant to disease diagnosis.

## Introduction

Modern neuroimaging techniques have enabled us to model the human brain as a brain connectivity network or a connectome. Rather than vector-based feature representations as traditional data, brain networks are inherently in the form of graph representations which are composed of brain regions as the nodes, *e.g.*, *insula*, *hippocampus*, *thalamus*, and functional/structural connectivities between the brain regions as the links. The linkage structure in these brain networks can encode tremendous information concerning the integrated activity of the human brain. For example, in brain networks derived from functional magnetic resonance imaging (fMRI), connections/links can encode correlations between brain regions in functional activity, while structural links in diffusion tensor imaging (DTI) can capture white matter fiber pathways connecting different brain regions. The complex structures and the lack of vector representations within these graph data raise a challenge for data mining. An effective model for mining the graph data should be able to extract a set of subgraph patterns for further analysis. Motivated by such challenges, graph mining research problems, in particular graph classification, have received considerable attention in the last decade.

The graph classification problem has been studied extensively. Conventional approaches focus on mining discriminative subgraphs from graph view alone. This is usually feasible for applications like molecular graph analysis, where a large set of graph instances with labels are available. For brain network analysis, however, usually we only have a small number of graph instances, ranging from 30 to 100 brain networks [19]. In these applications, the information from the graph view alone may not be sufficient for mining important subgraphs. Commonly, however, in neurological studies, hundreds of clinical, serologic, and cognitive measures are available for each subject in addition to brain networks derived from the neuroimaging data [4, 5]. These measures comprise multiple side views. This supplemental information, which is generally ignored, may contain a plurality of side views to guide the process of subgraph mining in brain networks.

**Fig. 1:**
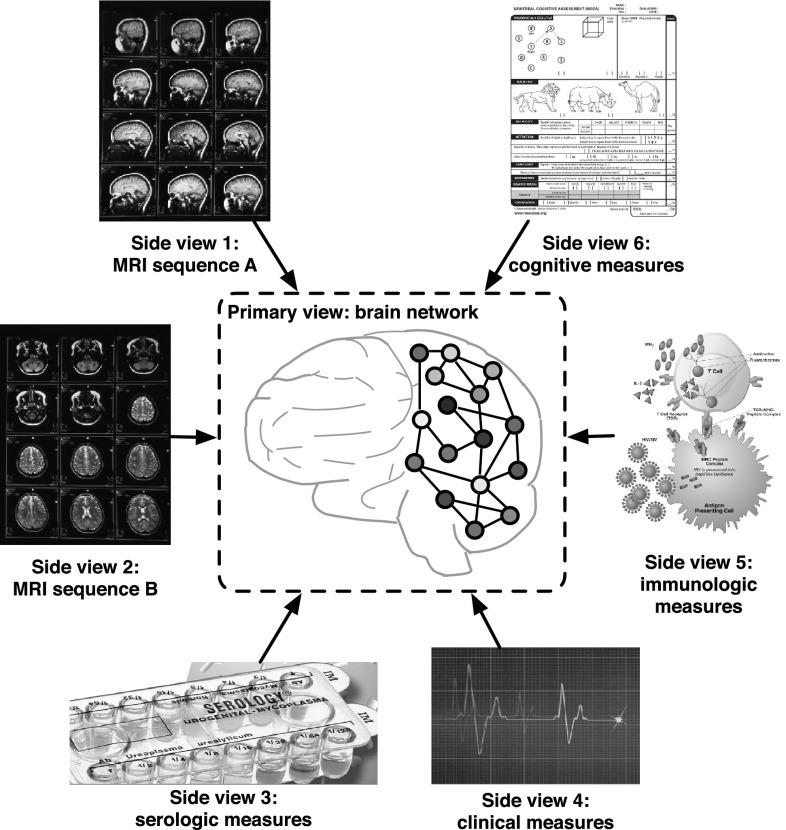
An example of multiple side views associated with brain networks in medical studies

Despite its value and significance, the feature selection problem for graph data using auxiliary views has not been studied in this context so far. There are two major difficulties in learning from multiple side views for graph classification, as follows:

### The primary view in graph representation

Graph data naturally compose the primary view for graph mining problems, from which we want to select discriminative subgraph patterns for graph classification. However, it raises a challenge for data mining with the complex structures and the lack of vector representations. Conventional feature selection approaches in vector spaces usually assume that a set of features are given before conducting feature selection. In the context of graph data, however, subgraph features are embedded within the graph structures and usually it is not feasible to enumerate the full set of subgraph features for a graph dataset before feature selection. Actually, the number of subgraph features grows exponentially with the size of graphs.

### The side views in vector representations

In many applications, side information is available along with the graph data and usually exists in the form of vector representations. That is to say, an instance is represented by a graph and additional vector-based features at the same time. It introduces us to the problem of how to leverage the relationship between the primary graph view and a plurality of side views, and how to facilitate the subgraph mining procedure by exploring the vector-based auxiliary views. For example, in brain networks, discriminative subgraph patterns for neurological disorders indicate brain injuries associated with particular regions. Such changes can potentially express in other medical tests of the subject, *e.g.*, clinical, immunologic, serologic, and cognitive measures. Thus, it would be desirable to select subgraph features that are consistent with these side views.

**Fig. 2:**
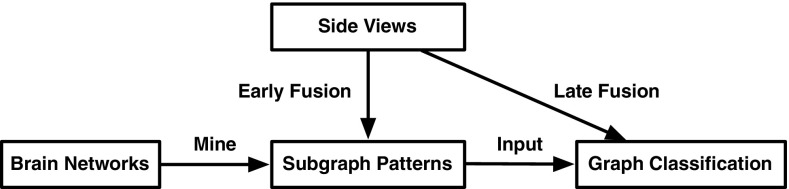
Two strategies of leveraging side views in feature selection process for graph classification: late fusion and early fusion [6]

Figure 2 illustrates two strategies of leveraging side views in the process of selecting subgraph patterns. Conventional graph classification approaches treat side views and subgraph patterns separately and may only combine them at the final stage of training a classifier. Obviously, the valuable information embedded in side views is not fully leveraged in the feature selection process. Most subgraph mining approaches focus on the drug discovery problem which have access to a great amount of graph data for chemical compounds. For neurological disorder identification, however, there are usually limited subjects with a small sample size of brain networks available. Therefore, it is critical to learn knowledge from other possible sources. We notice that transfer learning can borrow supervision knowledge from the source domain to help the learning on the target domain, *e.g.*, finding a good feature representation [10], mapping relational knowledge [24, 25], and learning across graph database [29]. However, to the best of our knowledge, they do not consider transferring complementary information from vector-based side views to graph database whose instances are complex structural graphs.

To solve the above problems, in this paper, we introduce a novel framework that fuses heterogeneous data sources at an early stage. In contrast to existing subgraph mining approaches that focus on a single view of the graph representation, our method can explore multiple vector-based side views to find an optimal set of subgraph features for graph classification. We first verify side information consistency via statistical hypothesis testing. Based on auxiliary views and the available label information, we design an evaluation criterion for subgraph features, named gSide. By deriving a lower bound, we develop a branch-and-bound algorithm, called gMSV, to efficiently search for optimal subgraph features with pruning, thereby avoiding exhaustive enumeration of all subgraph features. In order to evaluate our proposed model, we conduct experiments on graph classification tasks for neurological disorders, using fMRI and DTI brain networks. The experiments demonstrate that our subgraph selection approach using multiple side views can effectively boost graph classification performances. Moreover, we show that gMSV is more efficient by pruning the subgraph search space via gSide.

## Problem formulation

A motivation for this work is the premise that side information could be strongly correlated with neurological status. Before presenting the subgraph feature selection model, we first introduce the notations that will be used throughout this paper. Let ${\mathcal{D}}=\{G_1,\ldots ,G_n\}$ denote the graph dataset, which consists of *n* graph objects. The graphs within ${\mathcal{D}}$ are labeled by $[y_1,\ldots ,y_n]^\top$, where $y_i\in \{-1,+1\}$ denotes the binary class label of $G_i$.

### **Definition 1**

(Graph) A graph is represented as $G =(V,E)$, where $V=\{v_1,\ldots ,v_{n_v}\}$ is the set of vertices, $E\subseteq V\times V$ is the set of edges.

### **Definition 2**

(Subgraph) Let $G'=(V',E')$ and $G=(V,E)$ be two graphs. $G'$ is a subgraph of *G* (denoted as $G'\subseteq G$) iff $V'\subseteq V$ and $E'\subseteq E$. If $G'$ is a subgraph of *G*, then *G* is supergraph of $G'$.

### **Definition 3**

(Side view) A side view is a set of vector-based features ${\mathbf{z}}_i=[z_1,\ldots ,z_d]^\top$ associated with each graph object $G_i$, where *d* is the dimensionality of this view. A side view is denoted as ${\mathcal{Z}}=\{{\mathbf{z}}_1,\ldots ,{\mathbf{z}}_n\}$.

We assume that multiple side views $\{{\mathcal{Z}}^{(1)},\ldots ,{\mathcal{Z}}^{(v)}\}$ are available along with the graph dataset ${\mathcal{D}}$, where *v* is the number of side views. We employ kernels $\kappa ^{(p)}$ on ${\mathcal{Z}}^{(p)}$, such that $\kappa ^{(p)}_{ij}$ represents the similarity between $G_i$ and $G_j$ from the perspective of the *p*-th view. The RBF kernel is used as the default kernel in this paper, unless otherwise specified:

1 $$\kappa ^{(p)}_{ij}={\text{exp}} \left( -\frac{\Vert {\mathbf{z}}_i^{(p)}-{\mathbf{z}}_j^{(p)}\Vert _2^2}{d^{(p)}}\right)$$

In this paper, we adopt the idea of subgraph-based graph classification approaches, which assume that each graph object $G_j$ is represented as a binary vector ${\mathbf{x}}_j=[x_{1j},\ldots ,x_{mj}]^\top$ associated with the full set of subgraph patterns $\{g_1,\ldots ,g_m\}$ for the graph dataset $\{G_1,\ldots ,G_n\}$. Here $x_{ij}\in \{0,1\}$ is the binary feature of $G_j$ corresponding to the subgraph pattern $g_i$, and $x_{ij}=1$ iff $g_i$ is a subgraph of $G_j$ ($g_i\subseteq G_j$), otherwise $x_{ij}=0$. Let $X=[x_{ij}]^{m\times n}$ denote the matrix consisting of binary feature vectors using ${\mathcal{S}}$ to represent the graph dataset ${\mathcal{D}}$. $X=[{\mathbf{x}}_1,\ldots ,{\mathbf{x}}_n]=[{\mathbf{f}}_1,\ldots ,{\mathbf{f}}_m]^\top \in \{0,1\}^{m\times n}$. The full set ${\mathcal{S}}$ is usually too large to be enumerated. There is usually only a subset of subgraph patterns ${\mathcal{T}}\subseteq {\mathcal{S}}$ relevant to the task of graph classification. We briefly summarize the notations used in this paper in Table 1.

The key issue of discriminative subgraph selection using multiple side views is how to find an optimal set of subgraph patterns for graph classification by exploring the auxiliary views. This is non-trivial due to the following problems:

- How to leverage the valuable information embedded in multiple side views to evaluate the usefulness of a set of subgraph patterns?
- How to efficiently search for the optimal subgraph patterns without exhaustive enumeration in the primary graph space?

In the following sections, we will first introduce the optimization framework for selecting discriminative subgraph features using multiple side views. Next, we will describe our subgraph mining strategy using the evaluation criterion derived from the optimization solution.

**Table 1:** Important notations

Symbol	Definition and description
|.|	Cardinality of a set
$\Vert . \Vert$	Norm of a vector
${\mathcal{D}}=\{G_1,\ldots ,G_n\}$	Given graph dataset, $G_i$ denotes the *i*-th graph in the dataset
${\mathbf{y}}=[y_1,\ldots ,y_n]^\top$	Class label vector for graphs in ${\mathcal{D}}$, $y_i\in \{-1,+1\}$
${\mathcal{S}}=\{g_1,\ldots ,g_m\}$	Set of all subgraph patterns in the graph dataset ${\mathcal{D}}$
${\mathbf{f}}_i=[f_{i1},\ldots ,f_{in}]^\top$	Binary vector for subgraph pattern $g_i$, $f_{ij}=1$ iff $g_i\subseteq G_j$, otherwise $f_{ij}=0$
${\mathbf{x}}_j=[x_{1j},\ldots ,x_{mj}]^\top$	Binary vector for $G_j$ using subgraph patterns in ${\mathcal{S}}$, $x_{ij}=1$ iff $g_i\subseteq G_j$, otherwise $x_{ij}=0$
$X=[x_{ij}]^{m\times n}$	Matrix of all binary vectors in the dataset, $X=[{\mathbf{x}}_1,\ldots ,{\mathbf{x}}_n]=[{\mathbf{f}}_1,\ldots ,{\mathbf{f}}_m]^\top \in \{0,1\}^{m\times n}$
${\mathcal{T}}$	Set of selected subgraph patterns, ${\mathcal{T}}\subseteq {\mathcal{S}}$
${\mathcal{I}}_{\mathcal{T}}\in \{0,1\}^{m\times m}$	Diagonal matrix indicating which subgraph patterns are selected from ${\mathcal{S}}$ into ${\mathcal{T}}$
*min_sup*	Minimum frequency threshold; frequent subgraphs are contained by at least *min_sup* $\times |{\mathcal{D}}|$ graphs
*k*	Number of subgraph patterns to be selected
$\lambda ^{(p)}$	Weight of the *p*-th side view (default: 1)
$\kappa ^{(p)}$	Kernel function on the *p*-th side view (default: RBF kernel)

**Table 2:** Demographic characteristics

	HIV	Control	*p*
Age (mean years $\pm$ SD)	33.3 $\pm$ 10.1	31.4 $\pm$ 8.9	0.45
Gender (% male)	89 %	76 %	0.22
Race (% white)	62 %	76 %	0.22
Education (% college)	81 %	90 %	0.29

## Data analysis

A motivation for this work is that the side information could be strongly correlated with the health state of a subject. Before proceeding, we first introduce real-world data used in this work and investigate whether the available information from side views has any potential impact on neurological disorder identification.

### Data collections

In this paper, we study the real-world datasets collected from the Chicago Early HIV Infection Study at Northwestern University [27]. The clinical cohort includes 56 HIV (positive) and 21 seronegative controls (negative). Demographic information is presented in Table 2. HIV and seronegative groups did not differ in age, gender, racial composition or education level. More detailed information about data acquisition can be found in [5]. The datasets contain functional magnetic resonance imaging (fMRI) and diffusion tensor imaging (DTI) for each subject, from which brain networks can be constructed, respectively.

For fMRI data, we used DPARSF toolbox^1^ to extract a sequence of responds from each of the 116 anatomical volumes of interest (AVOI), where each AVOI represents a different brain region. The correlations of brain activities among different brain regions are computed. Positive correlations are used as links among brain regions. For details, functional images were realigned to the first volume, slice timing corrected, and normalized to the MNI template and spatially smoothed with an 8-mm Gaussian kernel. The linear trend of time series and temporally band-pass filtering (0.01–0.08 Hz) were removed. Before the correlation analysis, several sources of spurious variance were also removed from the data through linear regression: (i) six parameters obtained by rigid body correction of head motion, (ii) the whole-brain signal averaged over a fixed region in atlas space, (iii) signal from a ventricular region of interest, and (iv) signal from a region centered in the white matter. Each brain is represented as a graph with 90 nodes corresponding to 90 cerebral regions, excluding 26 cerebellar regions.

For DTI data, we used FSL toolbox^2^ to extract the brain networks. The processing pipeline consists of the following steps: (i) correct the distortions induced by eddy currents in the gradient coils and use affine registration to a reference volume for head motion, (ii) delete non-brain tissue from the image of the whole head [15, 30], (iii) fit the diffusion tensor model at each voxel, (iv) build up distributions on diffusion parameters at each voxel, and (v) repetitively sample from the distributions of voxel-wise principal diffusion directions. As with the fMRI data, the DTI images were parcellated into 90 regions (45 for each hemisphere) by propagating the Automated Anatomical Labeling (AAL) to each image [34]. Min-max normalization was applied on link weights.

In addition, for each subject, hundreds of clinical, imaging, immunologic, serologic, and cognitive measures were documented. Seven groups of measurements were investigated in our datasets, including *neuropsychological tests*, *flow cytometry*, *plasma luminex*, *freesurfer*, *overall brain microstructure*, *localized brain microstructure*, *brain volumetry*. Each group can be regarded as a distinct view that partially reflects subject status, and measurements from different medical examinations can provide complementary information. Moreover, we preprocessed the features by min-max normalization before employing the RBF kernel on each view.

**Table 3:** Hypothesis testing results (*p* values) to verify side information consistency

Side views	fMRI dataset	DTI dataset
Neuropsychological tests	1.3220e−20	3.6015e−12
Flow cytometry	5.9497e−57	5.0346e−75
Plasma luminex	9.8102e−06	7.6090e−06
Freesurfer	2.9823e−06	1.5116e−03
Overall brain microstructure	1.0403e−02	8.1027e−03
Localized brain microstructure	3.1108e−04	5.7040e−04
Brain volumetry	2.0024e−04	1.2660e−02

### Verifying side information consistency

We study the potential impact of side information on selecting subgraph patterns via statistical hypothesis testing. Side information consistency suggests that the similarity of side view features between instances with the same label should have higher probability to be larger than that with different labels. We use hypothesis testing to validate whether this statement holds in the fMRI and DTI datasets.

For each side view, we first construct two vectors ${\mathbf{a}}_s^{(p)}$ and ${\mathbf{a}}_d^{(p)}$ with an equal number of elements, sampled from the sets ${\mathcal{A}}_s^{(p)}$ and ${\mathcal{A}}_d^{(p)}$, respectively:

2 $${\mathcal{A}}_s^{(p)}=\{\kappa ^{(p)}_{ij}|y_iy_j=1\}$$

3 $${\mathcal{A}}_d^{(p)}=\{\kappa ^{(p)}_{ij}|y_iy_j=-1\}$$

Then, we form a two-sample one-tail *t* test to validate the existence of side information consistency. We test whether there is sufficient evidence to support the hypothesis that the similarity score in ${\mathbf{a}}_s^{(p)}$ is larger than that in ${\mathbf{a}}_d^{(p)}$. The null hypothesis is $H_0: \mu _s^{(p)}-\mu _d^{(p)}\le 0$, and the alternative hypothesis is $H_1: \mu _s^{(p)}-\mu _d^{(p)}>0$, where $\mu _s^{(p)}$ and $\mu _d^{(p)}$ represent the sample means of similarity scores in the two groups, respectively.

The *t* test results, *p* values, are summarized in Table 3. The results show that there is strong evidence, with significance level $\alpha =0.05$, to reject the null hypothesis on the two datasets. In other words, we validate the existence of side information consistency in neurological disorder identification, thereby paving the way for our next study of leveraging multiple side views for discriminative subgraph selection.

## Multi-side-view discriminative subgraph selection

In this section, we address the first problem discussed in Sect. 2 by formulating the discriminative subgraph selection problem as a general optimization framework as follows:

4 $${\mathcal{T}}^*={\mathrm{argmin}}_{{\mathcal{T}}\subseteq {\mathcal{S}}}{\mathcal{F}}({\mathcal{T}}) \quad {\text{s.t.}} \,|{\mathcal{T}}|\le k$$

where $|\cdot |$ denotes the cardinality and *k* is the maximum number of feature selected. ${\mathcal{F}}({\mathcal{T}})$ is the evaluation criterion to estimate the score (can be the lower the better in this paper) of a subset of subgraph patterns ${\mathcal{T}}$. ${\mathcal{T}}^*$ denotes the optimal set of subgraph patterns ${\mathcal{T}}^*\subseteq {\mathcal{S}}$.

### Exploring multiple side views: gSide

Following the observations in Sect. 3.2 that the side view information is clearly correlated with the prespecified label information, we assume that the set of optimal subgraph patterns should have the following properties. The similarity/distance between instances in the space of subgraph features should be consistent with that in the space of a side view. That is to say, if two instances are similar in the space of the *p*-th view (i.e., a high $\kappa ^{(p)}_{ij}$ value), they should also be close to each other in the space of subgraph features (i.e., a small distance between subgraph feature vectors). On the other hand, if two instances are dissimilar in the space of the *p*-th view (i.e., a low $\kappa ^{(p)}_{ij}$ value), they should be far away from each other in the space of subgraph features (i.e., a large distance between subgraph feature vectors). Therefore, our objective function could be to minimize the distance between subgraph features of each pair of similar instances in each side view, and maximize the distance between dissimilar instances. This idea is formulated as follows:

5 $$\mathop {\text {argmin}}\limits_{ {\mathcal{T}}\subseteq{\mathcal{S}}}\frac{1}{2}\sum _{p=1}^v\lambda ^{(p)}\sum _{i,j=1}^n \Vert {\mathcal{I}}_{\mathcal{T}}{\mathbf{x}}_i-{\mathcal{I}}_{\mathcal{T}}{\mathbf{x}}_j\Vert ^2_2\Theta ^{(p)}_{ij}$$

where ${\mathcal{I}}_{\mathcal{T}}$ is a diagonal matrix indicating which subgraph features are selected into ${\mathcal{T}}$ from ${\mathcal{S}}$, $({\mathcal{I}}_{\mathcal{T}})_{ii}=1$ iff $g_i\in {\mathcal{T}}$, otherwise $({\mathcal{I}}_{\mathcal{T}})_{ii}=0$. The parameters $\lambda ^{(p)}\ge 0$ are employed to control the contributions from each view.

6 $$\Theta_{ij}^{(p)} = \left\{ \begin{array}{ll} \frac{1}{|{\mathcal{H}}^{(p)}|} \, &{}\,(i,j)\in {\mathcal{H}}^{(p)}\\ -\frac{1}{|{\mathcal{L}}^{(p)}|}\,&{}\,(i,j)\in {\mathcal{L}}^{(p)} \end{array} \right.$$

where ${\mathcal{H}}^{(p)}=\{(i,j)|\kappa ^{(p)}_{ij}\ge \mu ^{(p)}\}$, ${\mathcal{L}}^{(p)}=\{(i,j)|\kappa ^{(p)}_{ij}<\mu ^{(p)}\}$, and $\mu ^{(p)}$ is the mean value of $\kappa ^{(p)}_{ij}$, i.e., $\frac{1}{n^2}\sum _{i,j=1}^n\kappa ^{(p)}_{ij}$. This normalization is to balance the effect of similar instances and dissimilar instances.

Intuitively, Eq. (5) will minimize the distance between subgraph features of similar instance-pairs with $\kappa ^{(p)}_{ij}\ge \mu ^{(p)}$, while maximizing the distance between dissimilar instance-pairs with $\kappa ^{(p)}_{ij}<\mu ^{(p)}$ in each view. In this way, the side view information is effectively used to guide the process of discriminative subgraph selection. The fact verified in Sect. 3.2 that the side view information is clearly correlated with the prespecified label information can be very useful, especially in the semi-supervised setting.

With prespecified information for labeled graphs, we further consider that the optimal set of subgraph patterns should satisfy the following constraints: labeled graphs in the same class should be close to each other; labeled graphs in different classes should be far away from each other. Intuitively, these constraints tend to select the most discriminative subgraph patterns based on the graph labels. Such an idea has been well explored in the context of dimensionality reduction and feature selection [2, 32].

The constraints above can be mathematically formulated as minimizing the loss function:

7 $$\mathop {\text {argmin}}\limits_{ {\mathcal{T}}\subseteq{\mathcal{S}}}\frac{1}{2}\sum _{i,j=1}^n\Vert {\mathcal{I}}_{\mathcal{T}}{\mathbf{x}}_i-{\mathcal{I}}_{\mathcal{T}}{\mathbf{x}}_j\Vert ^2_2\Omega _{ij}$$

where

8 $$\Omega _{ij} = \left\{ \begin{array}{ll} \frac{1}{|{\mathcal{M}}|}\,&{}\,(i,j)\in {\mathcal{M}}\\ -\frac{1}{|{\mathcal{C}}|}\,&{}\,(i,j)\in {\mathcal{C}}\\ 0\,&{}\,{\text{otherwise}} \end{array} \right.$$

and ${\mathcal{M}}=\{(i,j)|y_i y_j=1\}$ denotes the set of pairwise constraints between graphs with the same label, and ${\mathcal{C}}=\{(i,j)|y_i y_j=-1\}$ denotes the set of pairwise constraints between graphs with different labels.

By defining matrix $\Phi \in {\mathbb{R}}^{n\times n}$ as

9 $$\Phi _{ij}=\Omega _{ij}+\sum _{p=1}^v\lambda ^{(p)}\Theta ^{(p)}_{ij}$$

we can combine and rewrite the function in Eq. (5) and Eq. (7) as

10 $$\begin{aligned} {\mathcal{F}}({\mathcal{T}})&=\frac{1}{2}\sum _{i=1}^n\sum _{j=1}^n \Vert {\mathcal{I}}_{\mathcal{T}}{\mathbf{x}}_i-{\mathcal{I}}_{\mathcal{T}}{\mathbf{x}}_j\Vert ^2_2\Phi _{ij} \\& = {\text{tr}} ({\mathcal{I}}^{\top }_{\mathcal{T}}X(D-\Phi )X^{\top }{\mathcal{I}}_{\mathcal{T}}) \\& = {\text{tr}} ({\mathcal{I}}^{\top }_{\mathcal{T}}XLX^{\top }{\mathcal{I}}_{\mathcal{T}}) \\&=\sum _{g_i\in {\mathcal{T}}}{\mathbf {f}}_i^\top L{\mathbf{f}}_i \end{aligned}$$

where ${\text{tr}} (\cdot )$ is the trace of a matrix, *D* is a diagonal matrix whose entries are column sums of $\Phi$, i.e., $D_{ii}=\sum _{j}\Phi _{ij}$, and $L=D-\Phi$ is a Laplacian matrix.

#### **Definition 4**

(gSide)Let $'{\mathcal{D}}=\{G_1,\ldots ,G_n\}$ denote a graph dataset with multiple side views. Suppose $\Phi$ is a matrix defined as Eq. (9), and *L* is a Laplacian matrix defined as $L=D-\Phi$, where *D* is a diagonal matrix, $D_{ii}=\sum _{j}\Phi _{ij}$. We define an evaluation criterion *q*, called gSide, for a subgraph pattern $g_i$ as

11 $$q(g_i)={\mathbf{f}}_i^\top L {\mathbf{f}}_i$$

where ${\mathbf{f}}_i=[f_{i1},\ldots ,f_{in}]^\top \in \{0,1\}^n$ is the indicator vector for subgraph pattern $g_i$, $f_{ij}=1$ iff $g_i\subseteq G_j$, otherwise $f_{ij}=0$. Since the Laplacian matrix *L* is positive semi-definite, for any subgraph pattern $g_i$, $q(g_i)\ge 0$.

Based on gSide as defined above, the optimization problem in Eq. (4) can be written as

12 $${\mathcal{T}}^*= \mathop {\text {argmin}}\limits_{ {\mathcal{T}}\subseteq{\mathcal{S}}}\sum _{g_i \in {\mathcal{T}}}q(g_i) \quad {\text{s.t.}}\,|{\mathcal{T}}|\le k$$

The optimal solution to the problem in Eq. (12) can be found by using gSide to conduct feature selection on a set of subgraph patterns in ${\mathcal{S}}$. Suppose the gSide values for all subgraph patterns are denoted as $q(g_1)\le \cdots \le q(g_m)$ in sorted order, then the optimal solution to the optimization problem in Eq. (12) is

13 $${\mathcal{T}}^*= {\cup }_{i=1}^k\{g_i\}$$

### Searching with a lower bound: gMSV

Now we address the second problem discussed in Sect. 2, and propose an efficient method to find the optimal set of subgraph patterns from a graph dataset with multiple side views.

A straightforward solution to the goal of finding an optimal feature set is the exhaustive enumeration, i.e., we could first enumerate all subgraph patterns from a graph dataset, and then calculate the gSide values for all subgraph patterns. In the context of graph data, however, it is usually not feasible to enumerate the full set of subgraph patterns before feature selection. Actually, the number of subgraph patterns grows exponentially with the size of graphs. Inspired by recent advances in graph classification approaches [7, 20, 21, 37], which nest their evaluation criteria into the subgraph mining process and develop constraints to prune the search space, we adopt a similar approach by deriving a different constraint based upon gSide.

By adopting the gSpan algorithm proposed by Yan and Han [38], we can enumerate all the subgraph patterns for a graph dataset in a canonical search space. In order to prune the subgraph search space, we now derive a lower bound of the gSide value:

#### **Theorem 1**

Given any two subgraph patterns $g_i,g_j\in {\mathcal{S}}$, $g_j$ is a supergraph of $g_i$, i.e., $g_i\subseteq g_j$. The gSide value of $g_j$ is bounded by ${\hat{q}}(g_i)$, i.e., $q(g_j)\ge {\hat{q}}(g_i)$. ${\hat{q}}(g_i)$ is defined as

14 $${\hat{q}}(g_i)\triangleq {\mathbf{f}}_i^\top {\hat{L}} {\mathbf{f}}_i$$

where the matrix ${\hat{L}}$ is defined as ${\hat{L}}_{pq}\,\triangleq \,\min (0,L_{pq})$.

#### *Proof*

According to Definition 4,

15 $$q(g_j) = {\mathbf{f}}_j^\top L {\mathbf{f}}_j=\sum _{p,q:G_p,G_q\in {\mathcal{G}} (g_j)}L_{pq}$$

where ${\mathcal{G}} (g_j)\,\triangleq\, \{G_k|g_j\subseteq G_k,1\le k\le n\}$. Since $g_i\,\subseteq\, g_j$, according to anti-monotonic property, we have ${\mathcal{G}} (g_j)\,\subseteq\, {\mathcal{G}} (g_i)$. Also ${\hat{L}}_{pq}\,\triangleq\, \min (0,L_{pq})$, we have ${\hat{L}}_{pq}\le L_{pq}$ and ${\hat{L}}_{pq}\le 0$. Therefore,

16 $$\begin{aligned} q(g_j)&=\sum _{p,q:G_p,G_q\in {\mathcal{G}} (g_j)}L_{pq}\ge \sum _{p,q:G_p,G_q\in {\mathcal{G}} (g_j)} {\hat{L}}_{pq} \\&\ge \sum _{p,q:G_p,G_q\in {\mathcal{G}} (g_i)} {\hat{L}}_{pq} = {\hat{q}}(g_i) \end{aligned}$$

Thus, for any $g_i\subseteq g_j$, $q(g_j)\ge {\hat{q}}(g_i)$. □

We can now nest the lower bound into the subgraph mining steps in gSpan to efficiently prune the DFS code tree. During the depth-first search through the DFS code tree, we always maintain the currently top-*k* best subgraph patterns according to gSide and the temporally suboptimal gSide value (denoted by $\theta$) among all the gSide values calculated before. If ${\hat{q}}(g_i)\ge \theta$, the gSide value of any supergraph $g_j$ of $g_i$ should be no less than ${\hat{q}}(g_i)$ according to Theorem 1, i.e., $q(g_j)\ge {\hat{q}}(g_i)\ge \theta$. Thus, we can safely prune the subtree rooted from $g_i$ in the search space. If ${\hat{q}}(g_i)<\theta$, we cannot prune this subtree since there might exist a supergraph $g_j$ of $g_i$ such that $q(g_j)<\theta$. As long as a subgraph $g_i$ can improve the gSide values of any subgraphs in ${\mathcal{T}}$, it is added into ${\mathcal{T}}$ and the least best subgraph is removed from ${\mathcal{T}}$. Then we recursively search for the next subgraph in the DFS code tree. The branch-and-bound algorithm gMSV is summarized in Algorithm 1.

## Experiments

In order to evaluate the performance of the proposed solution to the problem of feature selection for graph classification using multiple side views, we tested our algorithm on brain network datasets derived from neuroimaging, as introduced in Sect. 3.1.

### Experimental setup

To the best of our knowledge, this paper is the first work on leveraging side information in feature selection problem for graph classification. In order to evaluate the performance of the proposed method, we compare our method with other methods using different statistical measures and discriminative score functions. For all the compared methods, gSpan [38] is used as the underlying searching strategy. Note that although alternative algorithms are available [17, 18, 37], the search step efficiency is not the focus of this paper. The compared methods are summarized as follows:

- gMSV: The proposed discriminative subgraph selection method using multiple side views. Following the observation in Sect. 3.2 that side information consistency is verified to be significant in all the side views, the parameters in gMSV are simply set to $\lambda ^{(1)}=\cdots =\lambda ^{(v)}=1$ for experimental purposes. In the case where some side views are suspect to be redundant, we can adopt the alternative optimization strategy to iteratively select discriminative subgraph patterns and update view weights.
- gSSC: A semi-supervised feature selection method for graph classification based upon both labeled and unlabeled graphs. The parameters in gSSC are set to $\alpha =\beta =1$ unless otherwise specified [21].
- Discriminative Subgraphs (Conf, Ratio, Gtest, HSIC): Supervised feature selection methods for graph classification based upon confidence [12], frequency ratio [16–18], *G* test score [37], and HSIC [20], respectively. The top-k discriminative subgraph features are selected in terms of different discrimination criteria.
- Frequent Subgraphs (Freq): In this approach, the evaluation criterion for subgraph feature selection is based upon frequency. The top-k frequent subgraph features are selected.

We append the side view data to the subgraph-based graph representations computed by the above algorithms before feeding the concatenated feature vectors to the classifier. Another baseline that only uses side view data is denoted as MSV.

For a fair comparison, we used LibSVM [9] with linear kernel as the base classifier for all the compared methods. In the experiments, 3-fold cross validations were performed on balanced datasets. To get the binary links, we performed simple thresholding over the weights of the links. The *threshold* for fMRI and DTI datasets was 0.9 and 0.3, respectively.

**Fig. 3:**
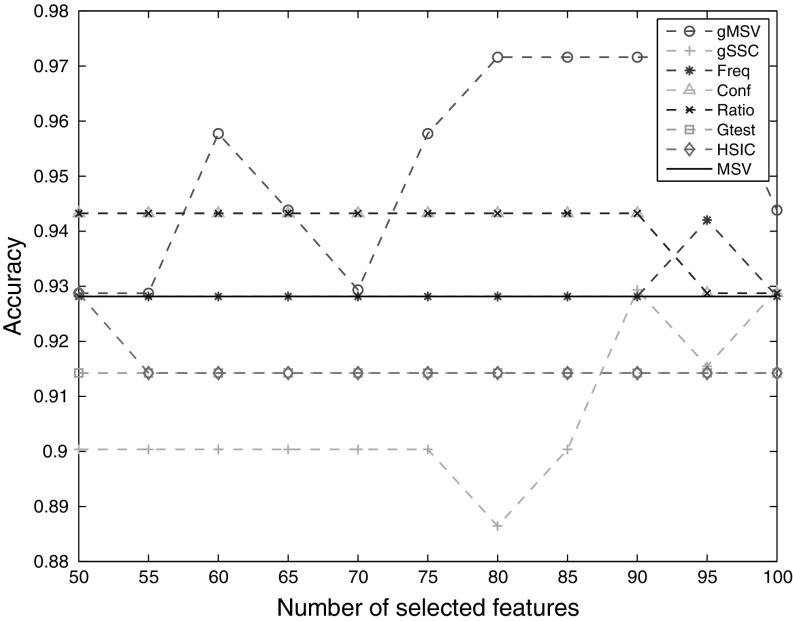
Classification performance on the fMRI dataset with different numbers of features.

**Fig. 4:**
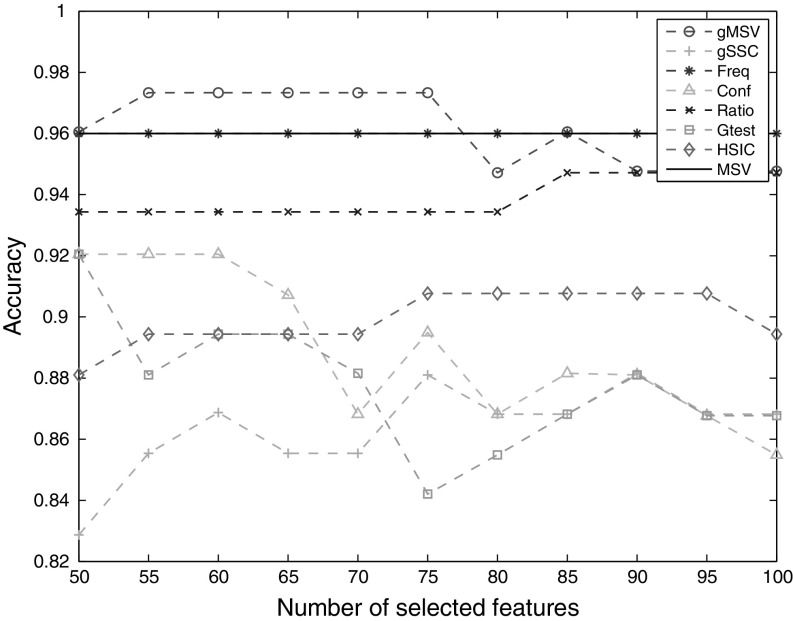
Classification performance on the DTI dataset with different numbers of features

### Performance on graph classification

The experimental results on fMRI and DTI datasets are shown in Figs. 3 and 4, respectively. The average performances with different numbers of features of each method are reported. Classification accuracy is used as the evaluation metric.

In Fig, 3, our method gMSV can achieve the classification accuracy as high as 97.16% on the fMRI dataset, which is significantly better than the union of other subgraph-based features and side view features. The black solid line denotes the method MSV, the simplest baseline that uses only side view data. Conf and Ratio can do slightly better than MSV. Freq adopts an unsupervised process for selecting subgraph patterns, resulting in a comparable performance with MSV, indicating that there is no additional information from the selected subgraphs. Other methods that use different discrimination scores without leveraging the guidance from side views perform even worse than MSV in graph classification, because they evaluate the usefulness of subgraph patterns solely based on the limited label information from a small sample size of brain networks. The selected subgraph patterns can potentially be redundant or irrelevant, thereby compromising the effects of side view data. Importantly, gMSV outperforms the semi-supervised approach gSSC which explores the unlabeled graphs based on the separability property. This indicates that rather than simply considering that unlabeled graphs should be separated from each other, it would be better to regularize such separability/closeness to be consistent with the available side views.

Similar observations are found in Fig. 4, where gMSV outperforms other baselines by achieving a good performance as high as 97.33% accuracy on the DTI dataset. We notice that only gMSV is able to do better than MSV by adding complementary subgraph-based features to the side view features. Moreover, the performances of other schemes are not consistent over the two datasets. The 2nd and 3rd best schemes, Conf and Ratio, for fMRI do not perform as well for DTI. These results support our premise that exploring a plurality of side views can boost the performance of graph classification, and the gSide evaluation criterion in gMSV can find more informative subgraph patterns for graph classification than subgraphs based on frequency or other discrimination scores.

**Fig. 5:**
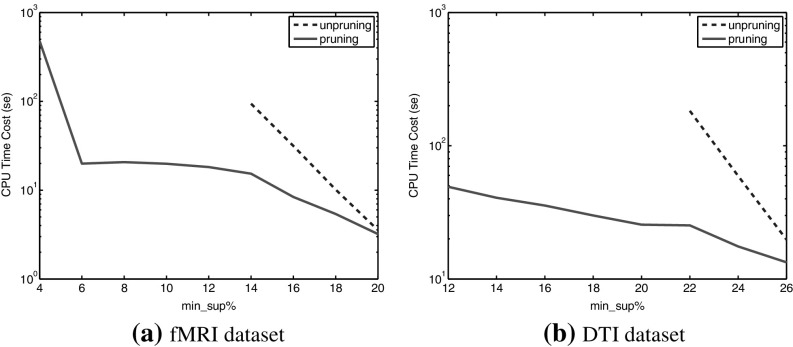
Average CPU time for pruning versus unpruning with varying min_sup

**Fig. 6:**
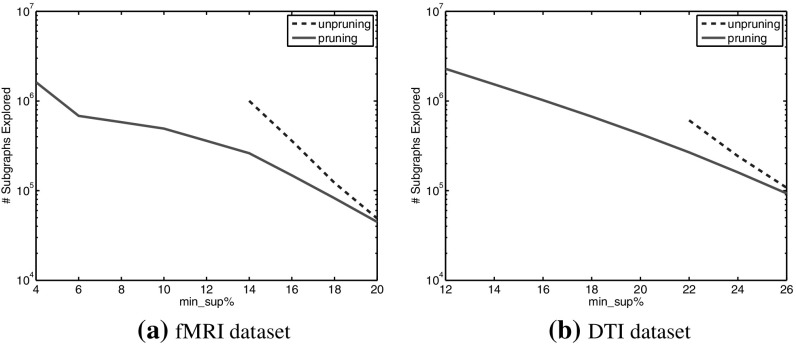
Average number of subgraph patterns explored in the mining procedure for pruning versus unpruning with varying min_sup

### Time and space complexity

Next, we evaluate the effectiveness of pruning the subgraph search space by adopting the lower bound of gSide in gMSV. In this section, we compare the runtime performance of two implementation versions of gMSV: the pruning gMSV uses the lower bound of gSide to prune the search space of subgraph enumerations, as shown in Algorithm  1; the unpruning gMSV denotes the method without pruning in the subgraph mining process, *e.g.*, deleting the line 13 in Algorithm  1. We test both approaches and recorded the average CPU time used and the average number of subgraph patterns explored during the procedure of subgraph mining and feature selection.

The comparisons with respect to the time complexity and the space complexity are shown in Figs. 5 and 6, respectively. On both datasets, the unpruning gMSV needs to explore exponentially larger subgraph search space as we decrease the *min_sup* value in the subgraph mining process. When the *min_sup* value is too low, the subgraph enumeration step in the unpruning gMSV can run out of the memory. However, the pruning gMSV is still effective and efficient when the *min_sup* value goes to very low, because its running time and space requirement do not increase as much as the unpruning gMSV by reducing the subgraph search space via the lower bound of gSide.

The focus of this paper is to investigate side information consistency and explore multiple side views in discriminative subgraph selection. As potential alternatives to the gSpan-based branch-and-bound algorithm, we could employ other more sophisticated searching strategies with our proposed multi-side-view evaluation criterion, gSide. For example, we can replace with gSide the *G* test score in LEAP [37] or the log ratio in COM [17] and GAIA [18], *etc.* However, as shown in Figs. 5 and 6, our proposed solution with pruning, gMSV, can survive at $min\_sup=4\%$; considering the limited number of subjects in medical experiments as introduced in Sect. 3.1, gMSV is efficient enough for neurological disorder identification where subgraph patterns with too few supported graphs are not desired.

### Effects of side views

In this section, we investigate contributions from different side views. The well-known precision, recall, and F1 are used as metrics. Precision is the fraction of positive predictions that are positive subjects. Recall is the fraction of positive subjects that are predicted as positive. F-measure is the harmonic mean of precision and recall. Table 4 shows performance of gMSV on the fMRI dataset by considering only one side view each time. In general, the best performance is achieved by simultaneously exploring all side views. Specifically, we observe that the side view *flow cytometry* can independently provide the most informative side information for selecting discriminative subgraph patterns on the fMRI brain networks. This is plausible as it implies that HIV brain alterations in terms of functional connectivity are most likely to express from this side view (i.e., in measures of immune function, the HIV hallmark). It is consistent with our finding in Sect. 3.2 that the side view *flow cytometry* is the most significantly correlated with the prespecified label information. Similar results on the DTI dataset are shown in Table 5.

**Table 4:** Average classification performances of gMSV on the fMRI dataset with different single-side views

Side views	Precision	Recall	F1
Neuropsychological tests	0.851	0.679	0.734
Flow cytometry	0.919	0.872	0.892
Plasma luminex	0.769	0.682	0.710
Freesurfer	0.851	0.737	0.785
Overall brain microstructure	0.824	0.500	0.618
Localized brain microstructure	0.686	0.605	0.637
Brain volumetry	0.739	0.737	0.731
All side views	1.000	0.949	0.973

**Table 5:** Average classification performances of gMSV on the DTI dataset with different single-side views

Side views	Precision	Recall	F1
Neuropsychological tests	0.630	0.705	0.662
Flow cytometry	0.847	0.808	0.822
Plasma luminex	0.801	0.705	0.744
Freesurfer	0.664	0.632	0.644
Overall brain microstructure	0.626	0.679	0.647
Localized brain microstructure	0.717	0.775	0.741
Brain volumetry	0.616	0.679	0.644
All side views	1.000	0.951	0.974

### Feature evaluation

**Fig. 7:**
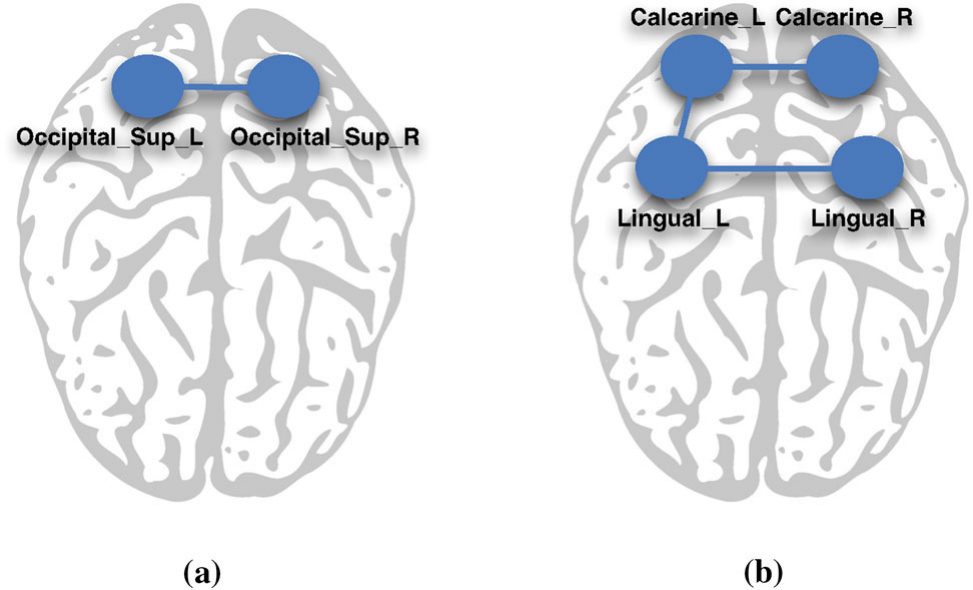
Discriminative subgraph patterns that are associated with HIV, selected from the fMRI dataset

**Fig. 8:**
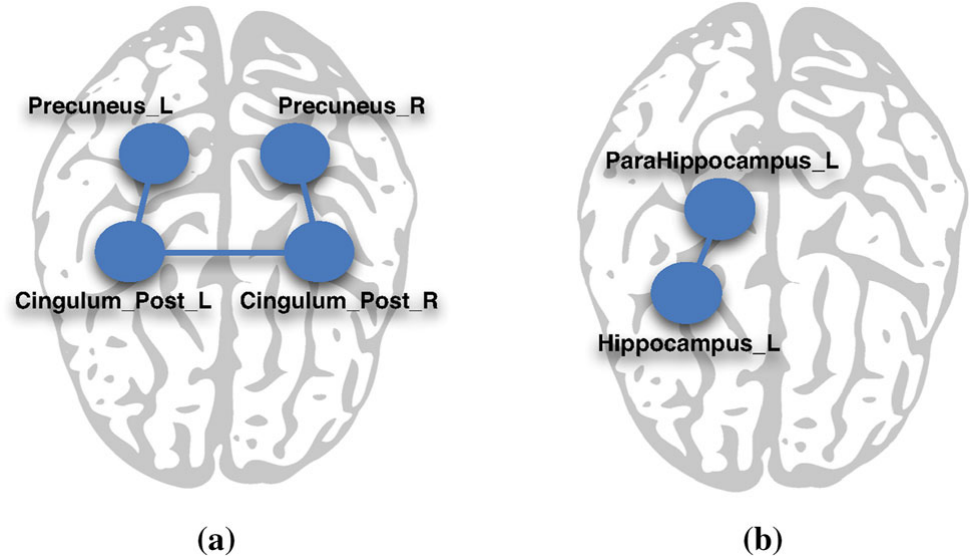
Discriminative subgraph patterns that are associated with HIV, selected from the DTI dataset

Figures 7 and 8 display the most discriminative subgraph patterns selected by gMSV from the fMRI dataset and the DTI dataset, respectively. These findings examining functional and structural networks are consistent with other in vivo studies [8, 35] and with the pattern of brain injury at autopsy [11, 23] in HIV infection. With the approach presented in this analysis, alterations in the brain can be detected in initial stages of injury and in the context of clinically meaningful information, such as host immune status and immune response (*flow cytometry*), immune mediators (*plasma luminex*) and cognitive function (*neuropsychological tests*). This approach optimizes the valuable information inherent in complex clinical datasets. Strategies for combining various sources of clinical information have promising potential for informing an understanding of disease mechanisms, for identification of new therapeutic targets and for discovery of biomarkers to assess risk and to evaluate response to treatment.

## Related work

To the best of our knowledge, this paper is the first work exploring side information in the task of subgraph feature selection for graph classification. Our work is related to subgraph mining techniques and multi-view feature selection problems. We briefly discuss both of them.

Mining subgraph patterns from graph data has been studied extensively by many researchers. In general, a variety of filtering criteria are proposed. A typical evaluation criterion is frequency, which aims at searching for frequently appearing subgraph features in a graph dataset satisfying a prespecified *min_sup* value. Most of the frequent subgraph mining approaches are unsupervised. For example, Yan and Han developed a depth-first search algorithm: gSpan [38]. This algorithm builds a lexicographic order among graphs, and maps each graph to an unique minimum DFS code as its canonical label. Based on this lexicographic order, gSpan adopts the depth-first search strategy to mine frequent-connected subgraphs efficiently. Many other approaches for frequent subgraph mining have also been proposed, *e.g.*, AGM [14], FSG [22], MoFa [3], FFSM [13], and Gaston [26].

Moreover, the problem of supervised subgraph mining has been studied in recent work which examines how to improve the efficiency of searching the discriminative subgraph patterns for graph classification. Yan et al. introduced two concepts *structural leap search* and *frequency-descending mining*, and proposed LEAP [37] which is one of the first works in discriminative subgraph mining. Thoma et al. proposed CORK which can yield a near-optimal solution using greedy feature selection [33]. Ranu and Singh proposed a scalable approach, called GraphSig, that is capable of mining discriminative subgraphs with a low frequency threshold [28]. Jin et al. proposed COM which takes into account the co-occurrences of subgraph patterns, thereby facilitating the mining process [17]. Jin et al. further proposed an evolutionary computation method, called GAIA, to mine discriminative subgraph patterns using a randomized searching strategy [18]. Our proposed criterion gSide can be combined with these efficient searching algorithms to speed up the process of mining discriminative subgraph patterns by substituting the *G* test score in LEAP [37] or the log ratio in COM [17] and GAIA [18], *etc.* Zhu et al. designed a diversified discrimination score based on the log ratio which can reduce the overlap between selected features by considering the embedding overlaps in the graphs [39]. Similar idea can be integrated into gSide to improve feature diversity.

There are some recent works on incorporating multi-view learning and feature selection. Tang et al. studied unsupervised multi-view feature selection by constraining that similar data instances from each view should have similar pseudo-class labels [31]. Cao et al. explored tensor product to bring different views together in a joint space and presents a dual method of tensor-based multi-view feature selection [4]. Aggarwal et al. considered side information for text mining [1]. However, these methods are limited in requiring a set of candidate features as input, and therefore are not directly applicable for graph data. Wu et al. considered the scenario where one object can be described by multiple graphs generated from different feature views and proposes an evaluation criterion to estimate the discriminative power and the redundancy of subgraph features across all views [36]. In contrast, in this paper, we assume that one object can have other data representations of side views in addition to the primary graph view.

In the context of graph data, the subgraph features are embedded within the complex graph structures and usually it is not feasible to enumerate the full set of features for a graph dataset before the feature selection. Actually, the number of subgraph features grows exponentially with the size of graphs. In this paper, we explore the side information from multiple views to effectively facilitate the procedure of discriminative subgraph mining. Our proposed feature selection for graph data is integrated to the subgraph mining process, which can efficiently prune the search space, thereby avoiding exhaustive enumeration of all subgraph features.

## Conclusion and future work

We presented an approach for selecting discriminative subgraph features using multiple side views. By leveraging available information from multiple side views together with graph data, the proposed method gMSV can achieve very good performance on the problem of feature selection for graph classification, and the selected subgraph patterns are relevant to disease diagnosis. This approach has broad applicability for yielding new insights into brain network alterations in neurological disorders and for early diagnosis.

A potential extension to our method is to combine fMRI and DTI brain networks to find discriminative subgraph patterns in the sense of both functional and structural connections. Other extensions include better exploring weighted links in the multi-side-view setting. It is also interesting to have our model applied to other domains where one can find graph data and side information aligned with the graph. For example, in bioinformatics, chemical compounds can be represented by graphs based on their inherent molecular structures and are associated with properties such as drug repositioning, side effects, ontology annotations. Leveraging all these information to find out discriminative subgraph patterns can be transformative for drug discovery.

## Footnotes

1 http://rfmri.org/DPARSF.

2 http://fsl.fmrib.ox.ac.uk/fsl/fslwiki.
